# Bio-Efficacy of Diatomaceous Earth, Household Soaps, and Neem Oil against *Spodoptera frugiperda* (Lepidoptera: Noctuidae) Larvae in Benin

**DOI:** 10.3390/insects12010018

**Published:** 2020-12-29

**Authors:** Crépin T. S. Aniwanou, Antonio A. C. Sinzogan, Jean M. Deguenon, Rachidatou Sikirou, David A. Stewart, Adam Ahanchede

**Affiliations:** 1 Laboratoire d’Entomologie Agricole (LEAg), Faculté des Sciences Agronomiques, Université d’Abomey-Calavi, 01 B.P. 526 Cotonou, Benin; sinzogan2001@yahoo.fr; 2 Department of Entomology and Plant Pathology, North Carolina State University, Campus Box 7647, 3230 Ligon Street, Raleigh, NC 27695, USA; jdeguen@ncsu.edu; 3 Laboratoire de Défense des Cultures (LDC), Centre de Recherches Agricoles d’Agonkanmey, Institut National des Recherches Agricoles du Bénin (INRAB), 01 B.P. 884 Cotonou, Benin; rachidatous@yahoo.fr; 4 Imerys Filtration Minerals, Inc., Roswell, GA 30076, USA; David.Stewart@imerys.com; 5 Laboratoire de Biologie Végétale, Faculté des Sciences Agronomiques, Université d’Abomey-Calavi, 01 B.P. 526 Cotonou, Benin; ahanchedeadam@yahoo.fr

**Keywords:** biopesticides, Palmida soap, Dezone, sustainable, fall armyworm, Benin

## Abstract

**Simple Summary:**

The fall armyworm (FAW) is an important pest native to the Americas which was recently introduced to Africa, where it has become a threat to maize production, a major food and cash crop. It is usually managed by chemical control. However, due to the drawbacks of large scale and indiscriminate use of synthetic chemical pesticides on human health and the environment, it is important to develop safer alternative methods. In a series of laboratory and field experiments conducted in Benin, West Africa, we evaluated the efficacy of several biorational products (soaps, detergents, diatomaceous earth, neem oil), and a semi-synthetic insecticide (emamectin benzoate) against fall armyworm larvae. Overall, the biorational insecticides provided similar and, in some cases, better control than the semi-synthetic insecticide used as positive control. The results suggest that the biorational insecticides tested in this study are cost-effective and may constitute viable control options for the fall armyworm.

**Abstract:**

*Spodoptera frugiperda* was first reported in Africa in 2016 and has since become a serious threat to maize/cereal production on the continent. Current control of the pest relies on synthetic chemical insecticides, which can negatively impact the environment and promote the development of resistance when used indiscriminately. Therefore, great attention is being paid to the development of safer alternatives. In this study, several biorational products and a semi-synthetic insecticide were evaluated. Two household soaps (“Palmida” and “Koto”) and a detergent (“So Klin”) were first tested for their efficacy against the larvae under laboratory conditions. Then, the efficacy of the most effective soap was evaluated in field conditions, along with PlantNeem (neem oil), Dezone (diatomaceous earth), and Emacot 19 EC (emamectin benzoate), in two districts, N’Dali and Adjohoun, located, respectively, in northern and southern Benin. The soaps and the detergent were highly toxic t second-instar larvae with 24 h lethal concentrations (LC_50_) of 0.46%, 0.44%, and 0.37% for So Klin, Koto, and Palmida, respectively. In field conditions, the biorational insecticides produced similar or better control than Emacot 19 EC. However, the highest maize grain yields of 7387 and 5308 kg/ha were recorded, respectively, with Dezone (N’Dali) and Emacot 19 EC (Adjohoun). A cost-benefit analysis showed that, compared to an untreated control, profits increased by up to 90% with the biorational insecticides and 166% with Emacot 19 EC. Therefore, the use of Palmida soap at 0.5% concentration, neem oil at 4.5 L/ha, and Dezone at 7.5 kg/ha could provide an effective, environmentally friendly, and sustainable management of *S. frugiperda* in maize.

## 1. Introduction

The fall armyworm (FAW) *Spodoptera frugiperda* J.E. Smith (Lepidoptera: Noctuidae), one of the most destructive lepidopteran pest species, has been recently reported in maize fields in Africa [1]. FAW is native to tropical and sub-tropical regions of the Americas and is an economically important pest that feeds on maize and other graminaceous plants [2]. It attacks all growth stages of maize and all aboveground plant structures [3] causing substantial foliar damage resulting in yield loss in the absence of control measures [4,5]. In 2016, more than 38,000 ha of maize production were damaged by FAW in northern Benin [1,6]. FAW has now been detected in over 40 African countries [7]. Several factors including host plants availability and certain environmental conditions enhance the abundance and persistence of the pest, which threatens the production of several key crops that provide livelihoods to millions of farmers in Africa [8].

Currently, FAW control is mainly achieved through the use of synthetic insecticides [9,10]. However, the indiscriminate use of chemical insecticides is often associated with adverse effects, such as pest resurgence, elimination of natural enemies, and presence of toxic residues in food, water, air, and soil, which can affect human health and disrupt the ecosystem [11]. Moreover, because of the biology and ecology of *S. frugiperda*, chemical insecticides used to control FAW are not always effective and insecticide resistance has been reported [12,13,14]. Therefore, great attention is being paid to promoting and or developing more effective and environmentally friendly alternative control methods [15].

Biorational substances offer a great source of insecticides that are effective against a wide range of insect pests and have relatively low environmental impacts. They include naturally derived active ingredients that are biodegradable, readily available and economically suitable for resource-limited smallholder farmers in Africa [16,17,18]. Diatomaceous earth (DE, fossils of phytoplankton called diatoms), for example, is a highly effective inert mechanical insecticide used in the management of stored-grain pests [19,20]. Constanski et al. [21] demonstrated the efficacy of DE applied alone and in combination with neem oil in the control of *S. eridania* and *S. frugiperda* larvae under laboratory conditions. Botanical extracts of various plant species, especially neem (*Azadirachta indica* A. Juss.), have also been reported to show insecticidal properties against FAW [22,23,24,25,26,27] in the laboratory. Soaps and detergents are also frequently reported in published surveys as local remedies used by farmers against the FAW. However, few studies have investigated the real-world applications of biorational insecticides on FAW in Africa, and, to the authors’ knowledge, no study has evaluated the effectiveness of DE in the field on the continent.

In the present study, the efficacy of natural insecticidal DE, locally available soaps and detergents, and neem oil against *S. frugiperda* was compared with that of the standard treatment, emamectin benzoate. A cost-benefit analysis was also conducted to assess the profitability of the different FAW management options tested.

## 2. Materials and Methods

### 2.1. Laboratory Efficacy of Locally Available Soaps and Detergents against FAW Larvae

#### 2.1.1. Experimental Conditions

Laboratory mortality assays were conducted at the Crop Protection Laboratory of the National Institute of Agricultural Research of Benin (INRAB). Experiments were carried out at 27 ± 3 °C, 70–75% relative humidity and a photoperiod of 12:12 h (Light:Dark). Temperature and humidity were recorded twice a week with HOBO data loggers (Onset Computer Corp., Bourne, MA, USA) placed in the laboratory.

#### 2.1.2. Insect Rearing

Second-instar larvae (5 days old) of *S. frugiperda* used in the laboratory assays were obtained from a laboratory colony. The colony was initially established with larvae collected from unsprayed maize fields at the research station of INRAB located in Abomey-Calavi, Benin. Different larval instars were collected from damaged maize plants at the vegetative stage (15–30 days old), transferred to the laboratory, and fed with tender and fresh maize leaves (sprouts) [28]. The pre-pupal stage was transferred to plastic cups (diameter: 14 cm, height: 10.5 cm) covered with tissue paper for pupation. Pupae were collected and placed in transparent plastic cups (diameter: 13.5 cm, height: 6.7 cm) used for adult emergence and as an oviposition device. Newly emerged adults were transferred to oviposition devices at a ratio of 6:4 (female: male), and sterile cotton soaked in 20% honey solution was placed inside the oviposition devices as a food source for emerging adults. The lid of the oviposition device was lined with wax paper as an oviposition substrate [29]. Two- to three-day-old egg batches were collected from the oviposition device and placed in a sterile plastic container (diameter: 14 cm, height: 10.5 cm) and covered with tissue paper. Egg hatch was monitored daily, and neonates were provided with diet made of maize shoots, and the tissue paper was replaced by a fine mesh lid. The diet was provided every 48 h throughout the larval stage. The insects were reared as described above until a sufficient second-generation population of *S. frugiperda* larvae was obtained to perform the experiments.

#### 2.1.3. Soaps and Detergent

“Palmida” and “Koto”, two local household soaps, as well as “So Klin” detergent (referred to as Klin in this paper), one of the most commonly used detergents in Benin, were tested. The “Palmida” soap (Palmida SA, Porto-Novo, Benin) used in this study results from the reaction of triglycerides (palm kernel oil, palm oil) with pure sodium hydroxide. Similarly, “Koto” soap (generally homemade), like most African black soaps, results from the reaction of triglycerides (palm oil, palm kernel oil) with potassium hydroxide (ashes from oil palm empty spadices or infructescences soaked in water).

Klin is composed of Linear alkyl benzene sulfonate, sodium tripolyphosphate, sodium carbonate, sodium sulphate, and enzymes (Natural Prime Resources Nigeria, Ltd. Agbara, Ogun State, Nigeria). These products were purchased at a local market in Cotonou, Benin.

#### 2.1.4. Bioassay

The different concentrations of soap and detergent solutions needed (g/mL) were prepared by dissolving the products in tap water 24 h prior to running the tests. The aqueous formulation of each product was evaluated at 0.25, 0.5, 0.75, 1, 1.5, and 2% concentrations, respectively, against second-instar larvae of *S. frugiperda* by immersing the larvae directly into the solutions for 5 s [30]. Palmida, Koto, and Klin were tested in separate trials, and treatments were arranged in a completely randomized design (CRD), with 10 replicates. Water was used as control in each trial, and controls 1, 2, and 3 relate to detergent Klin, Koto, and Palmida soap, respectively. Ten larvae were immersed for 5 s in each solution and transferred onto a tissue paper to remove excess liquid from the insect’s body surface. The larvae were then transferred separately into Petri dishes to avoid cannibalism. A 1 g portion of non-treated maize shoot was provided to each larva as food. The larvae used in each trial were obtained from the same rearing container. Larval mortality was recorded at 24 h post-exposure, and death was indicated by a failure to respond to mechanical stimulation [31].

### 2.2. Field Efficacy of Several Management Options against FAW

#### 2.2.1. Experimental Sites

The field studies were conducted from 27 August 2019 to 22 December 2019 at Ouénou village (09.46° N, 002.36° E), located in N’Dali district within the agro-ecological zone III (food crop region of South Borgou) in northern Benin, and from November 26, 2019 to 24 March 2020 at Lowé village (06.39° N, 002.27° E), located in Adjohoun district within the agro-ecological zone VIII (Region of fisheries and vegetables) in southern Benin. The southern regions in Benin are characterized by a bimodal rainfall pattern (rainy seasons in March–July and September–November), while the northern regions have a monomodal rainfall pattern (single rainy season from May to October) [32]. The annual rainfall ranges between 1000 and 1200 mm in N’Dali and between 950 and 1300 mm in Adjohoun. It is important to highlight that Adjohoun district is located in the Lower Valley of the Ouémé river and benefits from the flood recession period, during which several crops, including maize, are produced from November to January.

#### 2.2.2. Materials Tested

In field experiments, Palmida soap at 0.5% (based on the laboratory experiments and existing literature on soaps [33]), Emacot 19 EC (emamectin benzoate 19 g/L) at 0.6 L/ha (manufacturer’s recommendations), PlantNeem (100% neem oil) at 4.5 L/ha [34], and Dezone (85% silicon dioxide from diatoms) at 7.5 and 15 kg/ha were tested. Dezone was provided by Imerys (Imerys Filtration Minerals, Inc., Roswell, GA, USA) and applied as a wettable powder following manufacturer’s recommendations and based on previous studies [35]. Table 1 details the preparation of the insecticide solutions to treat 1 ha of maize.

#### 2.2.3. Experimental Design

Experiments were laid out in a randomized complete block design (RCBD) with 4 replicates. Each block contained 6 treatments (5 insecticide treatments (Table 1) + 1 untreated control). Plots treated with Emacot 19 EC were used as the positive control, while non-treated plots constituted the negative control. In the first experiment conducted at Ouénou (N’Dali), the treatments were applied to plots of 64 m^2^ (8 × 8 m) with a spacing of 2 m between plots and blocks. In the second experiment conducted at Lowé (Adjohoun), the experimental units were 16 m^2^ plots (4 × 4 m) with a spacing of 1 m between plots and blocks. Maize seeds, variety “Synée 2000” (extra-early maturing variety of 80 days with an expected yield of 2.5 t/ha) were sown with a spacing of 0.7 m between and within rows at Adjohoun and 0.8 m between rows and 0.4 m within rows at N’Dali. Three seeds were sown per plant hole and thinned to 2 plants per hole, 10 days after sowing (DAS). In the first experiment, the maize plants were provided with 200 kg of NPK fertilizer (N_14_P_23_K_14_S_5_B_1_) and 100 kg of urea/ha at 10 and 35 DAS, respectively. In the second experiment, no fertilizer was applied because of the alluvial nature of the soil in the region, which is known to be naturally very fertile [32] compared to a ferruginous tropical soil at Ouénou. Plots were manually kept weed-free throughout the experiments. All treatments (except the untreated control) were sprayed using a knapsack sprayer from 21 DAS to 42 DAS at 7-day intervals (4 applications), except for Emacot 19 EC, which was applied fortnightly (2 applications). Insecticides were applied during maize whorl stage. The first application was conducted at the early whorl stage (V6 stage), and the last application was performed at late whorl stage (V12) before the emergence of maize tassels [23]. Insecticides were sprayed from 5:00 to 6:30 p.m.

#### 2.2.4. Data Collection

Observations were performed before each spraying on 30 randomly selected plants per plot in the first experiment, while, in the second experiment, 15 randomly selected plants per plot were considered. The number of *S. frugiperda* larvae per plant (leaves and inside whorls) was recorded. Foliar damage severity was assessed by scoring each infested plant based on a rating scale adapted from Adéyè et al. [34], as follows: 0—Plant presenting no visible leaf-feeding damage (no damage); 1—Plant presenting superficial perforations and small circular holes or windowing effect (up to 1.3 cm in length); 2—Plant presenting large perforations (up to 2.5 cm in length); 3—Plant presenting a whorl with superficial perforations (up to 1.3 cm in length); 4—Plant presenting a whorl severely attacked (many elongated lesions with presence of larval droppings); and 5—Plant completely distorted or destroyed. The level of phytotoxicity from Palmida soap application was assessed following the description given by [36]: 0—Plant presenting no visible sign of toxicity; 1—Signs of yellowing to bronzing; 2—Wilting or curling; and 3—Necrosis and defoliation. At 60 DAS, plant height, stem thickness (root collar diameter), and leaf number were recorded. At physiological maturity, maize plants were harvested within three quadrats of 4 m^2^ per plot in the first experiment, while, in the second experiment, harvest was performed within one quadrat of 4 m^2^ per plot. Maize cobs and shelled grains (adjusted to 14% moisture) were weighed. Cob and grain yields were estimated in kg·ha^−1^, and the percentage reduction in the grain yield loss was calculated as follows:

(1) $$\%\mathrm{YR}=\frac{\mathrm{YT}-\mathrm{YC}}{\mathrm{YT}}*100,$$

where YT is the maize grain yield obtained in insecticide plots, and YC is the maize grain yield obtained in the untreated control plot.

### 2.3. Statistical Analyses

Concentration-mortality data from laboratory experiments were subjected to logistic regression analysis by Probit [37,38] to determine the median lethal concentration (LC_50_) of soap and detergent solutions tested. Abbott’s correction [39] was performed as part of the Probit procedure to correct for control mortality. The LC_50_ values were considered to be significantly different if the 95% confidence limits did not overlap. Generalized linear regression (with interaction) was used to assess the effect of sites and treatments on *S. frugiperda* larval populations per plant from the different collection dates. ANOVA was performed on the previous model using Pearson’s chi-square adjustment to determine the significance of the main factors and their interaction. Multiple comparisons among sites and treatments were performed using the Student-Newman-Keuls test (SNK, α = 0.05). Data related to the prevalence of infested plants (number of plants harboring larvae divided by the total number of plants sampled), as well as the percentage of damaged plants (number of plants with injury symptoms divided by the total number of plants sampled), were generated and submitted to a linear mixed effects model using the lme function of the nlme package [40]. Sites and treatments were analyzed as main factors and block as a random factor. Multiple comparisons among sites and treatments were performed using the Student-Newman-Keuls test. Leaf damage severity and phytotoxicity scores were analyzed using the Kruskal–Wallis test, and Dunn test was performed to separate the medians (α = 0.05). Data related to growth parameters, maize cob, and grain yields were subjected to two-way ANOVA. Plant height, stem thickness, maize cob, and grain yields were analyzed in a linear mixed effects model using the lme function of the nlme package [40], while leaf number was analyzed with the glmmTMB (generalized linear mixed models using template model builder) function of the glmmTMB package [41]. Sites and treatments were analyzed as fixed factors, while block was included as a random factor. Descriptive statistics (mean ± standard error) and Student-Newman-Keuls tests were performed to regroup homogeneous growth parameters by sites and treatments. Statistical analyses were performed using the R statistical software (Version 4.0.0, R Foundation for Statistical Computing, Vienna, Austria) (R Development Core Team 2020) [42].

### 2.4. Profitability Analysis

A cost comparison analysis was performed to assess the profitability of the different FAW management options. We considered that protectant products could be applied based on the number of sprayings performed in this study. Costs are related to expenditures on seeds, fertilizers, insecticides, machinery, water, protective clothing, and labor (weeding, fertilizer application, insecticide application, and harvesting) and do not take into account equipment amortization (Table 2).

## 3. Results

### 3.1. Larvicidal Activity of Soap and Detergent Solutions on Second-Instar Larvae of FAW in Laboratory

Mortality in the controls never exceeded 2%. The Probit analyses revealed that Palmida soap had the lowest LC_50_ (0.37%) and was more toxic to FAW larvae than Klin (0.46%) by a factor of 1.24 (Table 3 and File S1). No statistically significant difference was found between the LC_50_ values of the two soaps, but the LC_50_ of Palmida was 16% lower than that of Koto.

### 3.2. Field Efficacy of Dezone, Palmida Soap, and other Management Options Evaluated

#### 3.2.1. Impact of Insecticides Application on FAW Population

**Figure 1:**
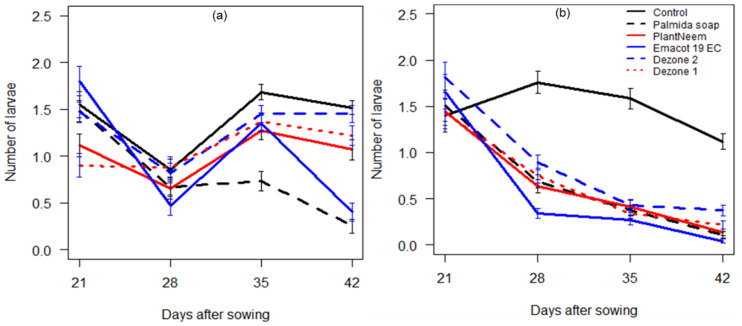
Mean numbers (±SE) of *Spodoptera frugiperda* larvae per plant based on weekly observations of 15 maize plants per plot at Adjohoun (**a**) and 30 plants at N’Dali (**b**) on each collection date. The treatments included Palmida soap, PlantNeem, Emacot 19 EC, Dezone, and the untreated control. Dezone 1 and 2 refer to application rates of 7.5 and 15 kg/ha, respectively.

The statistical analyses indicated significant differences in the average number of *S. frugiperda* larvae per plant, induced by factors days after sowing (Deviance = 416.71, df = 1, *p* < 0.001), sites (Deviance = 78.39, df = 1, *p* < 0.001), and treatments (Deviance = 269.48, df = 5, *p* < 0.001), as well as their interaction (Deviance = 68.95, df = 5, *p* < 0.001) (File S2). In general, the use of an insecticide significantly reduced the number of *S. frugiperda* larvae per maize plant. During this study, the number of larvae was higher in the untreated control compared to other treatments from 28 to 42 DAS at both sites (Figure 1). The average numbers of larvae per plant recorded with Dezone 2 and the untreated control were not significantly different at Adjohoun. However, the lowest overall average numbers of larvae per plant were obtained with Palmida soap at Adjohoun and with Emacot 19 EC at N’Dali (Figure 1).

#### 3.2.2. Prevalence of Infested Plants

**Figure 2:**
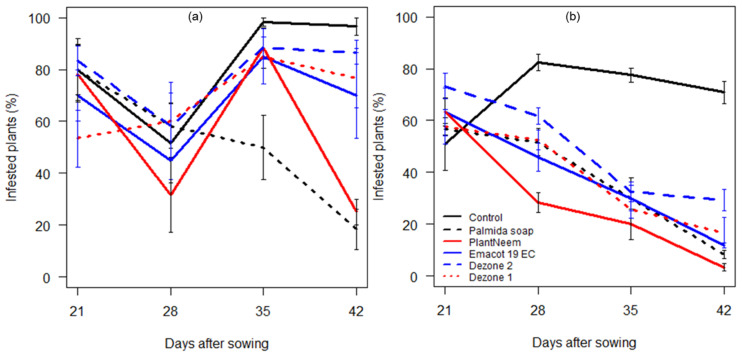
Percentage of plants harboring *Spodoptera frugiperda* larvae based on weekly observations of 15 maize plants per plot at Adjohoun (**a**) and 30 plants at N’Dali (**b**) on each collection date. The treatments included Palmida soap, PlantNeem, Emacot 19 EC, Dezone, and the untreated control. Dezone 1 and 2 refer to application rates of 7.5 and 15 kg/ha, respectively.

The statistical analyses indicated significant differences in the prevalence of infested plants, induced by factors sites (*F* = 54.06, df = 1, 165, *p* < 0.001), treatments (*F* = 16.84, df = 5, 165, *p* < 0.001), and days after sowing (*F* = 17.87, df = 1, 165, *p* < 0.001), as well as their interaction (*F* = 2.66, df = 5, 165, *p* = 0.024) (File S3). The prevalence of infested plants was, in general, higher in untreated plots compared to other treatments at both sites (Figure 2 and File S3). The lowest percentages of infested plants were achieved with Palmida soap and neem oil at Adjohoun (Figure 2a). The prevalence of infested plants decreased over time in plots treated with Emacot 19 EC, Palmida soap, neem oil, and Dezone at N’Dali (Figure 2b). Overall, FAW infestation was higher at Adjohoun compared to N’Dali.

#### 3.2.3. Impact of Insecticides Application on FAW Damage

FAW damage was evaluated with two parameters: the percentage of plants with typical FAW damage/injury symptoms, as well as damage severity.

**Figure 3:**
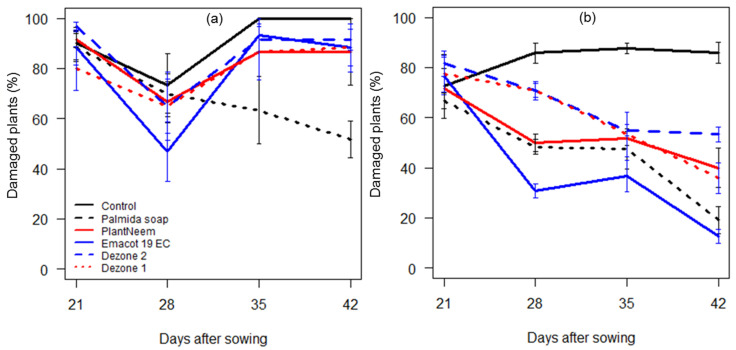
Percentage of plants with *Spodoptera frugiperda* damage based on weekly observations of 15 maize plants per plot at Adjohoun (**a**) and 30 plants at N’Dali (**b**) on each collection date. The treatments included Palmida soap, PlantNeem, Emacot 19 EC, Dezone, and the untreated control. Dezone 1 and 2 refer to application rates of 7.5 and 15 kg/ha, respectively.

The statistical analyses indicated significant differences in the percentage of damaged plants, induced by factors sites (*F* = 59.52, df = 1, 165, *p* < 0.001), treatments (*F* = 17.65, df = 5, 165, *p* < 0.001), and days after sowing (*F* = 12.61, df = 1, 165, *p* < 0.001), as well as their interaction (*F* = 3.18, df = 5, 165, *p* = 0.009) (File S4). Damaged plants were, in general, in higher numbers in the untreated plots compared to other treatments at both sites (Figure 3 and File S4). At Adjohoun, the lowest damage rates were achieved with Palmida soap (Figure 3a). FAW damage rates decreased over time in plots treated with Emacot 29 EC, Palmida soap, neem oil, and Dezone (Figure 3b). Overall, FAW damage rates were higher at Adjohoun compared to N’Dali.

**Figure 4:**
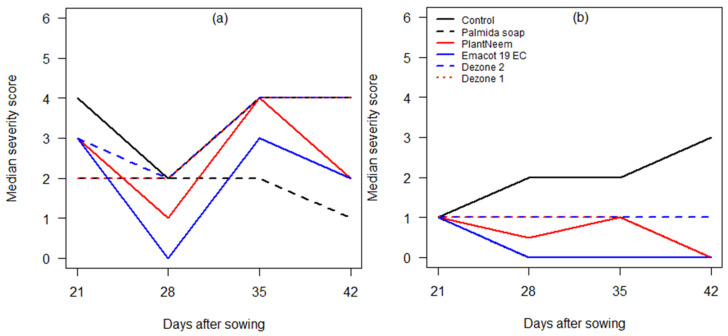
Effects of insecticide application on *S. frugiperda* larvae damage severity based on weekly observations of 15 maize plants per plot at Adjohoun (**a**) and 30 plants at N’Dali (**b**) on each collection date. Insect damage severity scores were adapted from Reference [34] and described in the Methods section. Dezone 1 and 2 refer to application rates of 7.5 and 15 kg/ha, respectively.

Plant damage severity varied significantly among treatments (Kruskal–Wallis chi square = 333.61, df = 5, *p* < 0.001) and between sites (Kruskal–Wallis chi square = 703.24, df = 1, *p* < 0.001) (File S5). Insect damage severity was higher in Adjohoun compared to N’Dali (Figure 4 and File S5). From 28 to 42 DAS, foliar damage was not different between Dezone applied at 15 kg/ha and the untreated control at Adjohoun (Figure 4a), while, at N’Dali, Palmida soap and Emacot 19 EC reduced damage to the same level (Figure 4b). However, the lowest median damage levels were recorded in plots treated with Emacot 19 EC and Palmida soap in both sites.

#### 3.2.4. Phytotoxicity Level Assessment

During this study, only Palmida soap induced signs of phytotoxicity (File S6). In general, the median phytotoxicity score exhibited by this treatment was null at both sites. However, the phytotoxicity induced by Palmida soap increased from 35 to 42, with a median score of 1 recorded at 42 DAS.

#### 3.2.5. Impact of Treatments on Growth Parameters

The statistical analyses showed that sites (*F* = 109.48, df = 1, 1065, *p* < 0.001) and treatments (*F* = 15.36, df = 5, 1065, *p* < 0.001), as well as their interaction (*F* = 8.87, df = 5, 1065, *p* < 0.001), significantly affected plant height (File S7). Sites (*F* = 37.06, df = 1, 1065, *p* < 0.001), treatments (*F* = 18.10, df = 5, 1065, *p* < 0.001), and their interaction (*F* = 11.67, df = 5, 1065, *p* < 0.001) also affected stem thickness (File S7). Values of plant height and stem thickness were, in general, significantly higher in plots where an insecticide was used compared to the untreated control plots. The tallest plants and thickest stems were recorded in the Dezone treatments at N’Dali, while, in Adjohoun, the plants in Dezone applied at 7.5 kg/ha displayed the thickest stems (Table 4). However, the number of leaves present on plants was not significantly affected by treatments (Deviance = 7.45, df = 5, *p* = 0.189) at either site (Deviance = 2.70, df = 1, *p* = 0.100) (File S7).

#### 3.2.6. Yields and Percentage Reduction in Grain Yield Loss

Cob and grain yields were significantly affected by treatments (cob: *F* = 10.93, df = 5, 81, *p* < 0.001; grain: *F* = 10.87, df = 5, 81, *p* < 0.001) and sites (cob: *F* = 58.51, df = 1, 81, *p* < 0.001; grain: *F* = 78.22, df = 1, 81, *p* < 0.001) (File S8). The yields obtained at N’Dali were 16 to 123% higher than those of Adjohoun. In general, yields were significantly higher in plots treated with an insecticide compared to control plots. The highest average maize yields were obtained in the two Dezone treatments and Emacot 19 EC at N’Dali, but the grain yield obtained with Dezone 1 was 10% higher than that of the positive control Emacot 19 EC. In Adjohoun, the two Dezone treatments promoted higher yields compared to other biopesticides, while the highest yields of all treatments were obtained with Emacot 19 EC. All insecticides substantially reduced yield losses with the percentage reduction in grain yield loss varying between 20 and 60% across treatments and sites (Table 5).

#### 3.2.7. Cost-Benefit Comparison Analysis

In the field, Dezone at 15 kg/ha produced similar results to Dezone at 7.5 kg/ha and, thus, was not considered in the cost-benefit analysis. Profits per ha of the different maize protection strategies were different and all positive. Profits were higher in N’Dali than in Adjohoun. Regardless of the district/site, the highest profits were obtained with Palmida soap, Dezone, and Emacot 19 EC. The profit was substantially increased when an insecticide was used. The net gain (percentage increase in profit) induced by the use of insecticides ranged between 3 and 51% at N’Dali and between 28 and 166% at Adjohoun (Table 6 and File S9).

## 4. Discussion

In the present study, we evaluated the efficacy of several biorational insecticides and a semi-synthetic insecticide against the fall armyworm, *Spodoptera frugiperda*, in laboratory and field conditions. In general, the results showed high efficacy of the products tested.

In the laboratory, the two soaps (Palmida and Koto) and the detergent (So Klin) were highly toxic to second-instar larvae in 5 s direct immersion experiments. The concentrations required to provide 50% mortality (LC_50_) were low and varied between 0.37 and 0.46% (*w*/*v*) of product in the solution (Table 3). Palmida and Koto are two multi-purpose household soaps (laundry, dishwashing, body wash/care), while Klin is a common detergent found in Benin.

Palmida soap was the most toxic as it displayed the lowest LC_50_ (0.37%). The insecticidal properties of soaps and detergents have been known for a long period of time [36,45,46]. In 1787, soaps were used to deal with small and soft-bodied insect pests of plants, such as aphids, thrips, psyllids, whiteflies, scales, and even mites [22,47]. Lee et al. [30] tested five household soap solutions at 0.4% against adult spider mites, *Tetranychus urticae*, and reported over 90% mortality 24 h after dipping the mites in the solutions for 1 s. Even though the modes of action of soaps and detergents have not been completely understood, they are known to act as contact insecticides [48], inducing mortality by disrupting the permeability of insect cuticles and by causing asphyxiation through obstruction of the spiracles [49].

In field conditions, Palmida soap, along with two other biorational insecticides, neem oil and Dezone, as wells as positive and negative controls were tested against FAW larvae. As is often the case in field trials, and for some parameters measured in this study, insecticide performance varied from one site to another. These variations could be explained by several factors including differences in pest abundance, soil type, climatic conditions, and farming practices.

FAW infestation levels, as well as damage to maize plants, were significant at both sites. First, the overall average numbers of *S. frugiperda* larvae per plant obtained in our study (0.6–1.5 larvae across treatments and sites) are similar to those obtained by Adéyè et al. [34] but higher than the action thresholds of 1.7–2.5 larvae per 10 plants established by Jaramillo-Barrios et al. [50] in maize crops. Second, it has been reported that FAW infestations during the whorl stage may result in yield losses of up to 73% when 55 to 100% of the plants are infested [51]. In our study, the percentage of infested plants was high in the treatments, especially in the untreated controls, where it varied between 80 and >90% at Adjohoun and between 50 and 80% at N’Dali (Figure 2 and File S3). This parameter also varied between the treatments at each site (File S3). Third, in Africa, the current recommendations establish action thresholds of 20% (early whorl stage) and 40% (late whorl stage) of plants with typical FAW damage [23,52]. In our study, the percentage of damaged plants in the controls was beyond these action thresholds throughout the study and also varied significantly between the treatments (Figure 3 and File S4).

Palmida soap applied in water significantly reduced larval densities, prevalence of infested plants, and foliar damage due to FAW larvae compared to the untreated control. Furthermore, it provided better protection against the FAW and its damage at Adjohoun, while, in N’Dali, Palmida soap and Emacot 19 EC provided similar protection levels.

Even though the pesticidal activity of soaps (commercial insecticidal soaps and household soaps) has been shown previously, the real-world application (on-farm trials) of household soaps has received less attention from the scientific community for various reasons [30]. Their use as adjuvants, on the other hand, is well documented [30,53,54]. To the best of our knowledge, this is the second report on the field efficacy of a household soap used as a stand-alone application against FAW in Africa and the first time Palmida soap was tested in the field as an insecticide. In contrast, Alata samina soap, an African black soap (used in Ghana and Nigeria) tested at 0.133% was not effective against FAW larvae in the field [55]. The results obtained in this study are similar to those of Amin et al. [33], who successfully field-tested soap solutions at concentrations varying between 0.5 and 3% against sucking pests in cotton. In our study, phytotoxicity (yellowing of leaves) was noticed in the Palmida treatment. This is not uncommon and has been reported before [22,36,45,46]. However, it is worth noticing that the phytotoxicity level was low and did not negatively impact plant growth or yield compared to the controls. Soaps are also thought to provide a more favorable ecotoxicological profile than broad-spectrum synthetic insecticides [54,56]. More studies are needed to fully understand the potential of locally available materials, such as Palmida soap, as control tools for *S. frugiperda*. In fact, the fate of household soaps in the environment, soil, etc., and their effects on natural enemies or other non-target species need to be investigated before wider use.

The present study also demonstrated the efficacy of neem oil (PlantNeem). The literature on the use of neem-based products (*Azadirachta indica*) to control various pests is extensive [54,57]. Neem oil, for example, is reported to control over 400 pests, acting mainly as an insecticide, antifeedant, or growth disruptor [57,58]. Previous studies have shown the efficacy of neem against *S. frugiperda*. Sisay et al. [27] reported high levels of FAW control in laboratory and field conditions using a wettable powder of neem seeds applied in water at 5% (*w*/*v*). Several other studies also highlighted the efficacy of neem in the control of *S. frugiperda* [34,59]. Moreover, the efficacy of neem oil has been reported for other noctuids, including the African armyworm *Spodoptera exempta*, the lawn armyworm *Spodoptera mauritia*, and the cotton leafworm *Spodoptera littoralis* [18].

Dezone is another insecticide of natural origin tested in this study with promising results. It is an EPA (U.S. Environmental Protection Agency) registered and OMRI (Organic Materials Review Institute)-approved mechanical insecticide/miticide [35]. Dezone is a high efficacy, natural DE-containing product designed to provide a physical mode of action against a broad range of insect pests to mitigate chemical insecticide resistance [35,60]. In fact, DE kills insects by removing the protective lipid layer of the insect’s cuticle, thus leading to death by desiccation. Due to their mode of action, inert powders, such as DE, are expected to prevent the development of resistance in insects [21,60,61,62]. DE is generally regarded as safe and is one of the most used grain protectants [20,63,64,65,66,67,68]. Our findings are in agreement with those of Constanski et al. [21], who demonstrated the efficacy of DE against second-instar larvae of *S. eridania* and *S. frugiperda* under laboratory conditions. Dezone was also effective against thrips, *Frankliniella fusca*, both in lab [69] and in cotton fields, at application rates similar to the ones used in this study [35]. In addition to reducing FAW infestations and damage levels in the field, plant growth was enhanced with Dezone applied at 7.5 kg/ha. This treatment also produced the highest yields at N’Dali with up to 38 and 9% in yield loss reductions compared to the negative and positive controls, respectively. In fact, it has been shown that spraying agricultural crops with mineral crop protectants reduces herbivory and oviposition, as well as water stress [70,71]. Furthermore, Dezone is rich in amorphous silicon dioxide (85% SiO_2_, Showler et al. [60]), and silicon has been characterized as a “quasi essential” mineral which promotes growth and increases yield in many plant species through various mechanisms, such as protection against biotic (diseases) and abiotic (metal ion toxicity and salinity) stresses [72,73]. However, doubling the application rate of Dezone (7.5 vs. 15 kg/ha) did not significantly improve the efficacy of the product. Mitchell et al. [35] did not observe significantly improved thrips control in cotton either when the dose was tripled (~7 vs. 22 kg/ha). These observations suggest that more research is needed to determine the optimum dose range for Dezone as a stand-alone system for FAW control and confirm its potential role as a growth and yield stimulator in maize fields.

Emacot 19 EC performed well and was used in this study as a positive control because it contains emamectin benzoate, one of the pesticidal molecules registered in South Africa for the control of *S. frugiperda* [74]. It is also registered in Benin [75] and is frequently used by farmers in Benin to control lepidopterous insects, including the FAW [76]. Emamectin benzoate is a semi-synthetic derivative of the natural fermentation product abamectin produced by the soil-dwelling actinomycete *Streptomyces avermitilis* and is used globally for its insecticidal and acaricidal properties [77]. It has been reported to dissipate quickly when released into the environment and to be less toxic to beneficial insects and, therefore, is often regarded as a less harmful active compared to most broad-spectrum synthetic insecticides [78]. However, emamectin benzoate is classified as a moderately toxic/hazardous compound by the Word Health Organization [79], and the careless use of this product may pose environmental risks and cause health hazards for animals and humans. For example, emamectin benzoate has been shown to be cytotoxic to human lung cells [80] and highly toxic to the silkworm moth, freshwater fish, and some bees [78]. Therefore, actions need to be taken (sensitization and training) to make sure that farmers use this product adequately. The good performance of Emacot 19 EC in this study was expected given the fact that the efficacy of emamectin benzoate against caterpillars is well documented in many agricultural crops [55,81,82].

Grain yields in our study were, in general, higher than the expected yield of 2500 kg/ha and substantially higher than the national average yield of ~1500 kg/ha usually obtained by farmers in Benin [83]. These differences could be explained by the fact that, in Adjohoun, for example, the soil is very fertile to the point where no fertilization—a key factor in yield response—is needed. In N’dali, mineral fertilization was provided (even in the negative control) following the recommendations from the Benin National Institute of Agricultural Research, which is not always the case with resource-limited farmers. We also provided adequate plant protection, thus mitigating yield loss risks due to insect pests. Another reason is that the expected yield (2500 kg/ha in this case) is often lower than the attainable/potential yield, which can also vary based on environmental conditions and farming practices [84]. Moreover, the highest grain yields obtained in this study are similar to those of Abadassi [85], with up to ~8000 kg/ha using the maize variety TZBSR. Tovihoudji et al. [84] were also able to obtain up to ~4500 kg/ha in grain yield (maize variety DMR-ESR) using reduced mineral fertilizer and manure rates.

It could be argued that factors not considered in this study, such as other pests or diseases, natural enemies, etc., may have been involved, affecting the yield differentially, especially among the treated plots. This is a reasonable assumption. However, it appears that, in this study, those factors may have not had a major impact. For example, the possible presence of plant diseases or other insect pests in the fields did not prevent the untreated controls to produce high maize yields (discussed previously). In addition, because the biorational products tested here (soaps, DE, neem oil), as well as the positive control, have been reported to be generally safe to beneficial arthropods [36,53,58,63,78], we do not expect, at least in theory, the populations of natural enemies to be significantly different between those treatments (and therefore to be a key factor explaining the differences in maize yields). At each experimental site, all the treated plots were exposed to the same environmental conditions and subjected to the same farming practices, except for the type of insecticide applied. The statements/evidence provided earlier (Results and Discussion) clearly show that pest levels recorded in this study were able to cause significant damage and that pest damage also varied between the treatments, at levels that can cause the significantly different effects in the yield recorded among the treatments. However, because other pests, diseases, and natural enemies, etc., were not investigated in this study, the role of these factors and other factors (unknown to the authors at this juncture) as yield response factors cannot be excluded. Additional studies are needed to help elucidate the role of other factors not considered in the current study in FAW management/yield response in maize.

Profitability was positive for all the treatments included in the cost-benefit analysis including the control. A legitimate concern could be that the overall higher cost of neem oil (US$ 97) and Dezone (US$ 57) compared to that of Emacot 19 EC (US$ 10) (File S9) could prevent the adoption of these biorational insecticides. However, in Benin, neem extracts (seeds and leaves) are the most commonly used biorational products. In addition, only one application rate was tested in this study for neem oil, while no improved efficacy was observed when the application rate was doubled for Dezone. Therefore, it may be possible to obtain similar or better results with fewer applications or lower application rates. This would substantially reduce insecticide costs, as well as total production costs, and further increase profits. Furthermore, Palmida soap, another biorational product tested in this study, was applied 4 times but only cost $5 more than Emacot 19 EC, which was applied twice ($15 vs. $10) (File S9). Finally, with the need for the world’s agricultural system to gradually move towards biorational pesticides, the results from our study could help fill the lack of biological pesticides availability on the market.

Overall, profitability was higher in the treatments where an insecticide was applied with increases in profitability, compared to the negative control, varying between 3% (PlantNeem) and 51% (Dezone at 7.5 kg/ha) at N’Dali and between 28% (PlantNeem) and 166% (Emacot 19 EC) at Adjohoun. The higher profits obtained in Adjohoun were mainly justified by lower production costs in this district. These results demonstrate the importance of using an insecticide in the management of *S. frugiperda* and suggest that biorational products, such as the ones tested in this study, are viable and cost-effective control methods.

## 5. Conclusions

In summary, this study investigated the bioactivity of several biorational insecticides against *Spodoptera frugiperda* larvae in laboratory and field conditions. In maize fields, all the biorational insecticides [PlantNeem, Palmida soap and Dezone (diatomaceous earth)] outperformed the untreated control and produced similar and, in some cases, better control than the chemical insecticide Emacot 19 EC. Furthermore, the cost-benefit analysis showed that spraying the plants with the biorational insecticides substantially increased profits. The findings from this study suggest that these insecticides may constitute viable control options for FAW management in maize. Additional studies are needed to better understand the potential of these technologies in FAW management.

## Figures and Tables

**Table 1:** Preparation of different insecticide solutions tested during the experiments.

Products ^a^	Dose/ha	Surfactant (Palmida Soap) kg	Volume of Application (L)
Dezone 1	7.5 kg	0.15	300
Dezone 2	15 kg	0.15	300
Palmida soap	1.5 kg	-	300
Emacot 19 EC	0.6 L	-	300
PlantNeem	4.5 L	0.6	300

^a^ Dezone 1 and 2 refer to application rates of 7.5 and 15 kg/ha, respectively.

**Table 2:** Costs of required items in maize production.

N°	Items	Units	Unit Price (US $) ^a^	Quantity/ha Per Crop Cycle
1	Maize seeds (Synée 2000)	Kg	1.6849	20
2	Water	L	0.0016 (0)	Variable
3	Fertilizer NPK	Bag of 50 kg	20.2190 (0)	4
4	Fertilizer Urea	Bag of 50 kg	20.2190 (0)	2
5	Knapsack sprayer (16 L) ^b^	Unit	50.5475	2
6	Protective clothing	Unit	8.4245	2
7	Emacot 19 EC	L	8.4245	1.2
8	Palmida soap	Bar	0.2527	Variable
9	PlantNeem	L	5.3917	18
10	Dezone	Kg	1.8888	30
11	Labor for weeding	Man day	5.0547 (3.3698)	8 (16)
12	Labor for fertilizer application	Man day	2.5273 (0)	12 (0)
13	Labor for spraying	Man day	8.4245 (3.3698)	Variable
14	Labor for harvesting	Man day	2.5273 (3.3698)	8 (12)

^a^ 1US $ = 593.5FCFA in average during the field studies [43]. Unit prices and quantities in parentheses only apply to the study conducted in Adjohoun. ^b^ The sprayer is typically used for several years. FCFA: Franc de la Communauté Financière Africaine. One kilogram of maize grain cost 150FCFA in local markets. The estimated price for Dezone is $34 per bag of 18 kg [44].

**Table 3:** Probit analysis and estimated median lethal concentration (LC_50_) values of Klin detergent and Koto and Palmida soaps on fall armyworm (FAW) second instars.

Soap/Detergent	N ^a^	Slope (SE ^b^)	LC_50_^c^ (95% CL ^d^)	χ^2 e^
Klin	700	1.84 (0.43)	0.462 (0.36–0.60)	2.22
Koto	700	1.84 (0.43)	0.443 (0.39–0.58)	3.19
Palmida	700	1.74 (0.44)	0.373 (0.27–0.51)	3.33

^a^ Values represent total number of larvae used including 100 control larvae. ^b^ SE = standard error. ^c^ Values represent percentage of product in the solution. Results were Abbott corrected. ^d^ Confidence limits. ^e^ Degrees of freedom for the χ^2^ values presented = 58 (File S1).

**Table 4:** Mean values (±SE) of plant height, stem thickness, and leaf number of maize under different treatments in the field.

Sites	Treatments	Growth Parameters ^a^		
		Plant Height (m)	Stem Thickness (cm)	Leaf Number
Adjohoun	Dezone 1	1.63 ± 0.04 ^ab^	3.45 ± 0.17 ^a^	11.32 ± 0.19 ^a^
	Dezone 2	1.58 ± 0.04 ^ab^	3.25 ± 0.17 ^ab^	11.07 ± 0.23 ^a^
	Emacot 19 EC	1.71 ± 0.03 ^a^	2.87 ± 0.07 ^bc^	11.42 ± 0.19 ^a^
	PlantNeem	1.51 ± 0.05 ^b^	3.17± 0.14 ^ab^	10.73 ± 0.23 ^a^
	Palmida soap	1.52 ± 0.03 ^b^	2.55 ± 0.06 ^cd^	11.30 ± 0.17 ^a^
	Control	1.62 ± 0.04 ^ab^	2.43 ± 0.07 ^d^	11.25 ± 0.18 ^a^
N’Dali	Dezone 1	1.89 ± 0.02 ^a^	2.81 ± 0.03 ^ab^	11.38 ± 0.15 ^a^
	Dezone 2	1.89 ± 0.02 ^a^	2.72 ± 0.04 ^a^	11.09 ± 0.15 ^a^
	Emacot 19 EC	1.75 ± 0.02 ^b^	2.65 ± 0.04 ^b^	10.73 ± 0.14 ^a^
	PlantNeem	1.75 ± 0.02 ^b^	2.78 ± 0.04 ^a^	10.94 ± 0.15 ^a^
	Palmida soap	1.71 ± 0.02 ^b^	2.80 ± 0.04 ^a^	10.85 ± 0.15 ^a^
	Control	1.62 ± 0.02 ^c^	2.45 ± 0.04 ^c^	9.99 ± 0.15 ^a^

^a^ Means within columns followed by different letters are significantly different, SNK multiple comparison test (*p* ≤ 0.05). Dezone 1 and 2 refer to application rates of 7.5 and 15 kg/ha, respectively.

**Table 5:** Maize cob and grain yields and yield loss reduction percentages under insecticide applications in the field.

Sites	Treatments	Yield (kg·ha^−1^) ^a^		Percentage Reduction in the Grain Yield Loss (%)
		Maize Cob	Maize Grain	
Adjohoun	Negative Control	3538 ± 322 ^b^	2060 ± 211 ^b^	-
	PlantNeem	4840 ± 1030 ^ab^	3618 ± 826 ^ab^	43
	Palmida soap	4975 ± 608 ^ab^	3830 ± 543 ^ab^	46
	Dezone 2	5298 ± 953 ^ab^	3968 ± 924 ^ab^	48
	Dezone 1	5928 ± 572 ^ab^	4408 ± 357 ^ab^	53
	Emacot 19 EC	7143 ± 533 ^a^	5308 ± 565 ^a^	61
N’Dali	Negative control	5718 ± 901 ^b^	4612 ± 766 ^b^	-
	PlantNeem	7163 ± 724 ^ab^	5804 ± 657 ^ab^	21
	Palmida soap	8056 ± 859 ^a^	6577 ± 754 ^ab^	30
	Emacot 19 EC	8312 ± 1065 ^a^	6694 ± 1016 ^a^	31
	Dezone 2	8073 ± 591 ^a^	6712 ± 520 ^a^	31
	Dezone 1	9003 ± 868 ^a^	7387 ± 735 ^a^	38

^a^ Means within columns followed by different letters are significantly different, SNK multiple comparison test (*p* ≤ 0.05). Dezone 1 and 2 refer to application rates of 7.5 and 15 kg/ha, respectively.

**Table 6:** Profitability of different FAW control strategies in maize production.

District	Treatments	Grain Yields (Kg/ha)	Average Revenue (US $ per ha) ^a^	Total Cost (US $ per ha)	Profit (US $ per ha)	Net Gain in Comparison to the Negative Control (%) ^b^
	Negative control	2060	520.64	128.05	392.59	-
	PlantNeem	3618	914.41	413.14	501.27	28
Adjohoun	Palmida soap	3830	967.99	325.18	642.81	64
	Dezone	4408	1114.08	368.20	745.88	90
	Emacot 19 EC	5308	1339.71	269.54	1043.16	166
	Negative control	4612	1165.62	245.99	919.62	-
	PlantNeem	5804	1466.89	519.59	947.29	3
N’Dali	Palmida soap	6577	1662.25	431.67	1230.58	34
	Emacot 19 EC	6694	1691.82	408.75	1283.06	40
	Dezone	7387	1866.97	474.69	1392.28	51

^a^ The revenue is obtained by using an estimated sale price of 0.25274US $ for 1 kg of maize grain; ^b^ %Net gain: percentage increase in profit due to the use of an insecticide.

## Data Availability

The data presented in this study are available from the corresponding author upon reasonable request.

## Supplementary Materials

The following are available online at: https://www.mdpi.com/2075-4450/12/1/18/s1, File S1: Probit analysis results, File S2: Generalized linear regression analyses on number of FAW larvae per plant, File S3: ANOVA results on the prevalence of infested plants, File S4: ANOVA results on the percentage of damaged plants, File S5: Kruskal–Wallis test performed on damage severity scores, File S6: Kruskal–Wallis test performed on phytotoxicity scores, File 7: ANOVA results on growth parameters, File S8: ANOVA results on maize cob and grain yields, File S9: Detailed profitability analyses in Adjohoun and N’Dali.

## References

[1] Goergen G., Kumar P.L., Sankung S.B., Togola A., Tamo M. (2016). First report of outbreaks of the fall armyworm *Spodoptera frugiperda* (J.E. Smith) (Lepidoptera, Noctuidae), a new alien invasive pest in West and Central Africa. PLoS ONE.

[2] Andrews K.L. (1980). The whorlworm, *Spodoptera frugiperda*, in central America and neighboring areas. Fla. Entomol..

[3] Buntin G.D. (1986). A review of plant response to fall armyworm, *Spodoptera frugiperda* (J.E. Smith), injury in selected field and forage crops. Fla. Entomol..

[4] Cruz I., Turpin F.T. (1982). Effects of *Spodoptera frugiperda* on different growth stages of corn. Pesqui. Agropecu. Bras..

[5] De Almeida S.R., de Souza A.R.W., Vieira S.M.J., de Oliveira H.G., Holtz A.M. (2002). Biology review, occurrence and control of *Spodoptera frugiperda* (Lepidoptera: Noctuidae) in corn in Brazil. Biosci. J..

[6] IPPC (2016). Les dégâts causés par *Spodoptera frugiperda*. (The damage caused by *Spodoptera frugiperda*). IPPC Official Pest Report.

[7] Rwomushana I., Bateman M., Beale T., Beseh P., Cameron K., Chiluba M., Clottey V., Davis T., Day R., Early R. (2018). Fall Armyworm: Impacts and Implications for Africa; Evidence Note Update.

[8] FAO Fall Armyworm Outbreak, a Blow to Prospects of Recovery for Southern Africa. http://www.fao.org/africa/news/detail-news/en/c/469532/.

[9] Cook D.R., Leonard B.R., Gore J. (2004). Field and laboratory performance of novel insecticides against armyworms (Lepidoptera: Noctuidae). Fla. Entomol..

[10] Agboyi L.K., Mensah S.A., Clottey V.A., Beseh P., Glikpo R., Rwomushana I., Day R., Kenis M. (2019). Evidence of leaf consumption rate decrease in Fall armyworm, *Spodoptera frugiperda*, larvae parasitized by *Coccygidium luteum*. Insects.

[11] Devine G.J., Furlong M.J. (2007). Insecticide use: Contexts and ecological successions. Agric. Hum. Values.

[12] Yu S.J. (1992). Detection and biochemical characterization of insecticide resistance in fall armyworm (Lepidoptera: Noctuidae). J. Econ. Entomol..

[13] Al-Sarar A., Hal F.R., Downer R.A. (2006). Impact of spray application methodology on the development of resistance to cypermethrin and spinosad by fall armyworm *Spodoptera frugiperda* (J.E. Smith). Pest Manag. Sci..

[14] Bateman M.L., Day R.K., Luke B., Edgington S., Kuhlmann U., Cock M.J.W. (2018). Assessment of potential biopesticide options for managing fall armyworm (*Spodoptera frugiperda*) in Africa. J. Appl. Entomol..

[15] FAO Integrated Management of the Fall Armyworm on Maize: A Guide for Farmer Field Schools in Africa. http://www.fao.org/3/I8665EN/i8665en.pdf.

[16] Rosell G., Quero C., Coll J., Guerrero A. (2008). Biorational insecticides in pest management. J. Pestic. Sci..

[17] Segnou J., Amougou A., Youmbi E., Njoya J. (2013). Effect of chemical treatments on pests and diseases of pepper (*Capsicum annuum* L.). Greener J. Agric. Sci..

[18] Duarte J.P., Redaelli L.R., Jahnke S.M., Trapp S. (2019). Effect of *Azadirachta indica* (Sapindales: Meliaceae) oil on *Spodoptera frugiperda* (Lepidoptera: Noctuidae) larvae and adults. Fla. Entomol..

[19] Shafighi Y., Ziaee M., Ghosta Y. (2014). Diatomaceous earth used against insect pests, applied alone or in combination with *Metarhizium anisopliae* and *Beauveria bassiana*. J. Plant Prot. Res..

[20] Kavallieratos N.G., Athanassiou C.G., Peteinatos G.G., Boukouvala M.C., Benelli G. (2018). Insecticidal effect and impact of fitness of three diatomaceous earths on different maize hybrids for the eco-friendly control of the invasive stored-product pest *Prostephanus truncatus* (Horn). Environ. Sci. Pollut. Res..

[21] Constanski K.C., Zorzetti J., Santoro P.H., Hoshino A.T., Neves P.M.O.J. (2016). Inert powders alone or in combination with neem oil for controlling *Spodoptera eridania* and *Spodoptera frugiperda* (Lepidoptera: Noctuidae) larvae. Ciênc. Agrár..

[22] Moore W.S., Profita J.C., Koehler C.S. (1979). Soaps for home landscape insect control. Calif. Agric..

[23] Prasanna B.M., Huesing J.E., Eddy R., Peschke V.M. (2018). Fall Armyworm in Africa: A Guide for Integrated Pest Management.

[24] Assefa F., Ayalew D. (2019). Status and control measures of fall armyworm (*Spodoptera frugiperda*) infestations in maize fields in Ethiopia: A review. Cogent Food Agric..

[25] Chimweta M., Nyakudya I.W., Jimu L., Mashingaidze A.B. (2019). Fall armyworm [*Spodoptera frugiperda* (J.E. Smith)] damage in maize: Management options for flood-recession cropping smallholder farmers. Int. J. Pest Manag..

[26] Harrison R.D., Thierfelder C., Baudron F., Chinwada P., Midega C., Schaffner U., Van Den Berg J. (2019). Agro-ecological options for fall armyworm (*Spodoptera frugiperda* J.E. Smith) management: Providing low cost, smallholder friendly solutions to an invasive pest. J. Environ. Manag..

[27] Sisay B., Tefera T., Wakgari M., Ayalew G., Mendesil E. (2019). The Efficacy of selected synthetic insecticides and botanicals against fall armyworm, *Spodoptera frugiperda*, in maize. Insects.

[28] Deryck P.W. (1979). Laboratory rearing of the fall armyworm. Fla. Entomol..

[29] Cruz I., Lourdes M., Silva D., Foster E. (2010). Efficiency of chemical pesticides to control *Spodoptera frugiperda* and validation of pheromone trap as a pest management tool in maize crop. Rev. Bras. Milho Sorgo.

[30] Lee C.-Y., Lo K.-C., Yao M.-C. (2006). Effects of household soap solutions on the mortality of the two-spotted spider mite, *Tetranychus urticae* Kock (Acari: Tetranychidae). Formos. Entomol..

[31] Williams K.A., Green D.W.J., Pascoe D., Gower D.E. (1986). The acute toxicity of cadmium to different larval stages of *Chrionomus riparius* (Diptera: Chironomidae) and its ecological significance for pollution regulation. Oecologia.

[32] Akossou A.Y.J., Attakpa E.Y., Fonton N.H., Sinsin B., Bosma R.H. (2016). Spatial and temporal analysis of maize (*Zea mays*) crop yields in Benin from 1987 to 2007. Agric. For. Meteorol..

[33] Amin M.R., Tithi D.A., Azad H.M.S., Hossain S.M.A. (2008). Evaluation of soap solution for management of cotton sucking pests at different locations of Bangladesh. J. Sci. Technol..

[34] Adéyè A.T., Sikirou R., Boukari S., Aboudou M., Amagnidé G.Y.G.A., Idrissou B.S., Idrissou-Touré M., Zocli B. (2018). Protection of maize crop against *Spodoptera frugiperda* with insecticides PlantNeem, Lambdace 25 EC and Viper 46 EC and yield loss reduction in Benin. J. Rech. Sci. Univ. Lomé.

[35] Mitchell R.D., Mott D.W., Dhammi A., Reisig D.D., Roe R.M., Stewart D. Field evaluation of a new thrips control agent for cotton: A mechanical insecticide. Proceedings of the 2018 Beltwide Cotton Conferences.

[36] Curkovic T., Gill H.K., Goyal G. (2016). Detergents and soaps as tools for IPM in agriculture. Integrated Pest Management (IPM): Environmentally Sound Pest Management.

[37] Finney D.J. (1971). Probit Analysis.

[38] Savi M.K., Mangamana E.T., Jean Marcel Deguenon J.M., Hounmenou C.G., Kakaï R.G. (2017). Determination of lethal concentrations using an R software function integrating the Abbott correction. J. Agric. Sci. Technol..

[39] Abbott W.S. (1925). A method of computing the effectiveness of an insecticide. J. Econ. Entomol..

[40] Pinheiro J., Bates D., DebRoy S., Sarkar D., R Core Team nlme: Linear and Nonlinear Mixed Effects Models. R Package Version 3.1-147. https://CRAN.R-project.org/package=nlme&gt;/.

[41] Brooks M.E., Kristensen K., van Benthem K.J., Magnusson A., Berg C.W., Nielsen A., Skaug H.J., Machler M., Bolker B.M. (2017). glmmTMB balances speed and flexibility among packages for Zero-inflated Generalized Linear Mixed Modeling. R J..

[42] R Development Core Team (2016). R: A Language and Environment for Statistical Computing.

[43] OANDA. https://www1.oanda.com/currency/converter/.

[44] Stewart D.A. (2020). Personal communication.

[45] Osborne L.S. (1982). Is soap a viable method for controlling *Tetranychus urticae* on plants in the interior environment?. Proc. Fla. State Hort. Soc..

[46] Osborne L.S. (1984). Soap spray: An alternative to conventional acaricide for controlling the twospotted spider mite (Acari: Tetranychidae) in greenhouses. J. Econ. Entomol..

[47] Ware G.W. (2000). The Pesticide Book.

[48] Szumlas D.E. (2002). Behavioral responses and mortality in German cockroaches (Blattodea: Blatellidae) after exposure to dishwashing liquid. J. Econ. Entomol..

[49] Koehler C.S., Barclay L.W., Kretchun T.M. (1983). Soaps as insecticides. Calif. Agric..

[50] Jaramillo-Barrios C.I., Varón-Devia E.H., Monje-Andrade B. (2020). Economic injury level and action thresholds for *Spodoptera frugiperda* (J.E. Smith) (Lepidoptera: Noctuidae) in maize crops. Rev. Fac. Nac. Agron. Medellín.

[51] Kuate A.F., Hanna R., Fotio A.R.P.D., Abang A.F., Nanga S.N., Ngatat S., Tindo M., Masso C., Rose Ndemah R., Suh C. (2019). *Spodoptera frugiperda* Smith (Lepidoptera: Noctuidae) in Cameroon: Case study on its distribution, damage, pesticide use, genetic differentiation and host plants. PLoS ONE.

[52] FAO, PPD Manual on Integrated Fall Armyworm Management.

[53] Mkenda P., Mwanauta R., Stevenson P.C., Ndakidemi P., Mtei K., Belmain S.R. (2015). Extracts from field margin weeds provide economically viable and environmentally benign pest control compared to synthetic pesticides. PLoS ONE.

[54] Dougoud J., Toepfer S., Bateman M., Jenner W.H. (2019). Efficacy of homemade botanical insecticides based on traditional knowledge. A review. Agron. Sustain. Dev..

[55] Babendreier D., Agboyi L.K., Beseh P., Osae M., Nboyine J., Ofori S.E.K., Frimpong J.O., Clottey V.A., Kenis M. (2020). The efficacy of alternative, environmentally friendly plant protection measures for control of Fall armyworm, *Spodoptera frugiperda*, in maize. Insects.

[56] Liu T.X., Stansly P.A. (2000). Insecticidal activity of surfactants and oils against silverleaf whitefly (*Bemisia argentifolii*) nymphs (Homoptera: Aleyrodidae) on collards and tomato. Pest Manag. Sci..

[57] Isman M.B., Hall F.R., Menn J.J. (1999). Neem and related natural products. Biopesticides: Use and Delivery.

[58] Erler F., Cetin H., Saribasak H., Serttas A. (2010). Laboratory and field evaluations of some botanical pesticides against the cedar leaf moth, *Acleris undulana*. J. Pest Sci..

[59] Phambala K., Tembo Y., Kasambala T., Kabambe V.H., Stevenson P.C., Belmain S.R. (2020). Bioactivity of common pesticidal plants on Fall armyworm larvae (*Spodoptera frugiperda*). Plants.

[60] Showler A.T., Flores N., Caesar R.M., Mitchel R.D., De León A.A.P. (2020). Lethal effects of a commercial diatomaceous earth dust product on *Amblyomma americanum* (Ixodida: Ixodidae) larvae and nymphs. J. Med. Entomol..

[61] Deguenon J.M., Azondekon R., Agossa F.R., Padonou G.G., Anagonou R., Ahoga J., N’dombidje B., Akinro B., Stewart D.A., Wang B. (2020). Imergard^TM^WP: A non-chemical alternative for an indoor residual spray, effective against pyrethroid-resistant *Anopheles gambiae* (s.l.) in Africa. Insects.

[62] Deguenon J.M., Riegel C., Cloherty-Duvernay E.R., Stewart D.A., Wang B., Gittins D., Tihomirov L., Apperson C.S., McCord M.G., Roe R.M. (2020). New mosquitocide derived from volcanic rock. J. Med. Entomol..

[63] Korunic Z. (1998). Diatomaceous earths, a group of natural insecticides. J. Stored Prod. Res..

[64] Arthur F.H. (2000). Toxicity of diatomaceous earth to red flour beetles and confused flour beetles (Coleoptera: Tenebrionidae): Effects of temperature and relative humidity. J. Econ. Entomol..

[65] Fields P., Korunic Z. (2000). The effect of grain moisture content and temperature on the efficacy of diatomaceous earths from different geographical locations against stored-product beetles. J. Stored Prod. Res..

[66] Subramanyam B., Roesli R., Subramanyam B., Hagstrum D.W. (2000). Inert dusts. Alternatives to Pesticides in Stored-Product IPM.

[67] Athanassiou C.G., Kavallieratos N.G., Tsaganou F.C., Vayias B.J., Dimizas C.B., Buchelos C.T. (2003). Effect of grain type on the insecticidal efficacy of SilicoSec against *Sitophilus oryzae* (L.) (Coleoptera: Curculionidae). Crop. Prot..

[68] Athanassiou C.G., Kavallieratos N.G., Peteinatos G.G., Petrou S.E., Boukouvala M.C., Tomanovic Z. (2007). Influence of temperature and humidity on insecticidal effect of three diatomaceous earth formulations against larger grain borer (Coleoptera: Bostrychidae). J. Econ. Entomol..

[69] Zhu J., Dhammi A., Roe R.M., Stewart D. Novel mechanical pesticides for tobacco thrips, *Frankliniela fusca*, control in cotton. Proceedings of the 2016 Beltwide Cotton Conferences.

[70] Glenn D.M., Puterka G.J., Drake S.R., Unruh T.R., Knight A.L., Baherle P., Prado E., Baugher T.A. (2001). Particle film application influences apple leaf physiology, fruit yield, and fruit quality. J. Am. Soc. Hortic. Sci..

[71] Lapointe S.L., Mckenzie C.L., Hall D.G. (2006). Reduced oviposition by *Diaprepes abbreviates* (Coleoptera: Curculionidae) and growth enhancement of citrus by surround particle film. J. Econ. Entomol..

[72] Epstein E. (1999). Silicon. Annu. Rev. Plant Physiol. Plant Mol. Biol..

[73] Ma J.F., Mitani N., Nagao S., Konishi S., Tamai K., Iwashita T., Yano M. (2004). Characterization of the silicon uptake and molecular mapping of the silicon transporter gene in rice. Plant Physiol..

[74] Bezuidenhout S.R., Nunkumar A. Chemical Control Options for Fall Armyworm in Maize. Research & Technology Bulletin (2016–2017). https://www.kzndard.gov.za.

[75] Comité National de Gestion des Pesticides (CNGP). https://zoomagro.com/wp-content/uploads/2020/03/Liste-actialis%C3%A9e-des-pesticides-autoris%C3%A9s_F%C3%A9vrier-2020-1.pdf.

[76] Houngbo S., Zannou A., Aoudji A., Sinzogan A., Sossou C.H., Sikirou R., Zossou E., Totin Vodounon S.H., Adomou A., Ahanchede A. (2020). Farmers’ knowledge and management practices of fall armyworm, *Spodoptera frugiperda* J.E. Smith in Benin. Agriculture.

[77] Jansson R.K., Dybas R.A., Ishaava I., Degheele D. (1998). Avermectins: Biochemical mode of action, biological activity and agricultural importance. Insecticides with Novel Modes of Action-Mechanisms and Application.

[78] Deng L., Chen L., Guan S., Liu J., Liang J., Li X., Li Z. (2020). Dissipation of emamectin benzoate residues in rice and rice-growing environments. Molecules.

[79] Wang L., Zhao P., Zhang F., Li Y., Du F., Pan C. (2012). Dissipation and residue behavior of emamectin benzoate on apple and cabbage field application. Ecotoxicol. Environ. Saf..

[80] Niu C., Wang C., Wu C., Yang J., Yanan Wen Y., Meng S., Lin X., Pang X., An L. (2020). Toxic effects of the emamectin benzoate exposure on cultured human bronchial epithelial (16HBE) cells. Environ. Pollut..

[81] Shivalingaswamy T.M., Kumar A., Satpathy S., Rai A.B. (2008). Efficacy of emamectin benzoate in the management of vegetable pests. Prog. Hortic..

[82] Gacemi A., Guenaoui Y. (2012). Efficacy of emamectin benzoate on *Tuta absoluta* Meyrick (Lepidoptera: Gelechiidae) infesting a protected tomato crop in Algeria. Acad. J. Entomol..

[83] Soglo Y.Y., Nonvide G.A.N. (2019). Climate change perceptions and responsive strategies in Benin: The case of maize farmers. Clim. Chang..

[84] Tovihoudji P.G., Akponikpè P.B.I., Agbossou E.K., Bielders C.L. (2019). Variability in maize yield and profitability following hill-placement of reduced mineral fertilizer and manure rates under smallholder farm conditions in northern Benin. Field Crops Res..

[85] Abadassi J. (2001). Caractérisation de quelques variétés améliorées de maïs cultivées au Bénin. Bull. Rech. Agron..

