# Supplementary material for: Bio-Efficacy of Diatomaceous Earth, Household Soaps, and Neem Oil against Spodoptera frugiperda (Lepidoptera: Noctuidae) Larvae in Benin
Source: Insects. 2020 Dec 29;12(1):18. doi: 10.3390/insects12010018 (PMC7823957; doi:10.3390/insects12010018)
Supplement: Supplementary file 1 [file insects-12-00018-s001.zip › insects-984553-s-XML/SUPPLEMENTARY MATERIALS_UPDATED/File S1_Probit analysis results.docx]

**File S1 : Probit analysis results**

Klin detergent

###########

Deviance Residuals:

Min 1Q Median 3Q Max

-0.52502 -0.08886 0.08392 0.17479 0.44852

Coefficients:

Estimate Std. Error z value Pr(>|z|)

(Intercept) 1.4195 0.3272 4.339 1.43e-05 ***

log(Concentration) 1.8408 0.4269 4.312 1.62e-05 ***

---

Signif. codes: 0 ‘***’ 0.001 ‘**’ 0.01 ‘*’ 0.05 ‘.’ 0.1 ‘ ’ 1

(Dispersion parameter for binomial family taken to be 1)

Null deviance: 33.4994 on 59 degrees of freedom

Residual deviance: 2.6538 on 58 degrees of freedom

AIC: 27.8

Number of Fisher Scoring iterations: 7

###### Wald significance test

----------

Chi-squared test:

X2 = 21.8, df = 2, P(> X2) = 1.9e-05

############ Pearson's chi-squared test

Df Deviance Resid. Df Resid. Dev Pr(>Chi)

NULL 59 33.499

log(Concentration) 1 30.846 58 2.654 2.794e-08

m0$chi_square

[1] 2.21626

############

LD SE LCL UCL

p = 0.50: 0.462 1.143 0.356 0.601

p = 0.90: 0.928 1.180 0.671 1.284

p = 0.95: 1.130 1.220 0.765 1.670

Koto soap

Deviance Residuals:

Min 1Q Median 3Q Max

-0.56248 -0.09928 0.07510 0.15893 0.45202

Coefficients:

Estimate Std. Error z value Pr(>|z|)

(Intercept) 1.4949 0.3411 4.383 1.17e-05 ***

log(Concentration) 1.8374 0.4316 4.258 2.07e-05 ***

---

Signif. codes: 0 ‘***’ 0.001 ‘**’ 0.01 ‘*’ 0.05 ‘.’ 0.1 ‘ ’ 1

(Dispersion parameter for binomial family taken to be 1)

Null deviance: 33.8712 on 59 degrees of freedom

Residual deviance: 3.8815 on 58 degrees of freedom

AIC: 25.99

Number of Fisher Scoring iterations: 7

###### Wald significance test

----------

Chi-squared test:

X2 = 21.5, df = 2, P(> X2) = 2.1e-05

############ Pearson's chi-squared test

Df Deviance Resid. Df Resid. Dev Pr(>Chi)

NULL 59 33.871

log(Concentration) 1 29.99 58 3.881 4.343e-08 ***

m0$chi_square

[1] 3.186496

###########

LD SE LCL UCL

p = 0.50: 0.443 1.147 0.339 0.580

p = 0.90: 0.890 1.181 0.642 1.234

p = 0.95: 1.085 1.222 0.733 1.606

Palmida soap

############# Résumé

Deviance Residuals:

Min 1Q Median 3Q Max

-0.57552 -0.12771 0.05822 0.12966 0.85169

Coefficients:

Estimate Std. Error z value Pr(>|z|)

(Intercept) 1.7213 0.3862 4.457 8.33e-06 ***

log(Concentration) 1.7441 0.4401 3.963 7.39e-05 ***

---

Signif. codes: 0 ‘***’ 0.001 ‘**’ 0.01 ‘*’ 0.05 ‘.’ 0.1 ‘ ’ 1

(Dispersion parameter for binomial family taken to be 1)

Null deviance: 29.4041 on 59 degrees of freedom

Residual deviance: 3.9574 on 58 degrees of freedom

AIC: 20.24

Number of Fisher Scoring iterations: 7

###### Wald significance test

----------

Chi-squared test:

X2 = 20.4, df = 2, P(> X2) = 3.7e-05

############ Pearson's chi-squared test

Df Deviance Resid. Df Resid. Dev Pr(>Chi)

NULL 59 29.4041

log(Concentration) 1 25.447 58 3.9574 4.548e-07 ***

m0$chi_square

[1] 3.327989

###########

LD SE LCL UCL

p = 0.50: 0.373 1.169 0.274 0.506

p = 0.90: 0.777 1.192 0.550 1.097

p = 0.95: 0.957 1.237 0.631 1.453
