# Supplementary material for: Bio-Efficacy of Diatomaceous Earth, Household Soaps, and Neem Oil against Spodoptera frugiperda (Lepidoptera: Noctuidae) Larvae in Benin
Source: Insects. 2020 Dec 29;12(1):18. doi: 10.3390/insects12010018 (PMC7823957; doi:10.3390/insects12010018)
Supplement: Supplementary file 1 [file insects-12-00018-s001.zip › insects-984553-s-XML/SUPPLEMENTARY MATERIALS_UPDATED/File S2_Generalized linear regression analyses on number of FAW larvae per plant.docx]

**File S2 : Generalized linear regression analyses on number of FAW larvae per plant**

Df Deviance Resid. Df Resid. Dev Pr(>Chi)

NULL 4319 5670.5

DAS 1 416.71 4318 5253.8 < 2.2e-16 ***

Sites 1 78.39 4317 5175.4 < 2.2e-16 ***

Treatments 5 269.48 4312 4906.0 < 2.2e-16 ***

DAS:Sites 1 201.63 4311 4704.3 < 2.2e-16 ***

DAS:Treatments 5 207.77 4306 4496.6 < 2.2e-16 ***

Sites:Treatments 5 85.79 4301 4410.8 < 2.2e-16 ***

DAS:Sites:Treatments 5 68.95 4296 4341.8 1.692e-13 ***

Signif. codes: 0 ‘***’ 0.001 ‘**’ 0.01 ‘*’ 0.05 ‘.’ 0.1 ‘ ’ 1

**Multiple comparison tests**

Adjohoun

> library(agricolae)

> S0=SNK.test(mode1,'Treatments');S0

$statistics

MSerror Df Mean CV

0.9823381 1434 1.1 90.1027

$parameters

Test name.t ntr alpha

SNK Treatments 6 0.05

$snk

Table CriticalRange

2 2.774149 0.1774821

3 3.317945 0.2122727

4 3.637423 0.2327119

5 3.862571 0.2471163

6 4.035558 0.2581835

$means

Larvae std r Min Max Q25 Q50 Q75

Dezone 1 1.0916667 0.8629601 240 0 3 0 1 2

Dezone 2 1.2958333 0.8181923 240 0 4 1 1 2

Emacot 19 EC 1.0041667 1.0490000 240 0 4 0 1 2

PlantNeem 1.0250000 0.8722836 240 0 4 0 1 2

Palmida soap 0.7833333 0.8550449 240 0 3 0 1 2

Control 1.4000000 0.8716838 240 0 4 1 2 2

$groups

Larvae groups

Control 1.4000000 a

Dezone 2 1.2958333 a

Dezone 1 1.0916667 b

PlantNeem 1.0250000 b

Emacot 19 EC 1.0041667 b

Palmida soap 0.7833333 c

N’Dali

> library(agricolae)

> S0=SNK.test(mode1,'Treatments');S0

$statistics

MSerror Df Mean CV

0.9428312 2874 0.821875 118.1439

$parameters

test name.t ntr alpha

SNK Treatments 6 0.05

$snk

Table CriticalRange

2 2.772975 0.1228973

3 3.316215 0.1469735

4 3.635286 0.1611146

5 3.860107 0.1710786

6 4.032818 0.1787331

$means

Larvae s td r Min Max Q25 Q50 Q75

Dezone 1 0.6833333 1.179033 480 0 10 0 0 1

Dezone 2 0.8791667 1.253797 480 0 10 0 0 1

Emacot 19 EC 0.5770833 1.277023 480 0 10 0 0 1

PlantNeem 0.6562500 1.091131 480 0 6 0 0 1

Palmida soap 0.6708333 1.193223 480 0 10 0 0 1

Control 1.4645833 1.422602 480 0 10 0 1 2

$groups

Larvae groups

Control 1.4645833 a

Dezone 2 0.8791667 b

Dezone 1 0.6833333 c

Palmida soap 0.6708333 c

PlantNeem 0.6562500 c

Emacot 19 EC 0.5770833 c
