# Supplementary material for: Bio-Efficacy of Diatomaceous Earth, Household Soaps, and Neem Oil against Spodoptera frugiperda (Lepidoptera: Noctuidae) Larvae in Benin
Source: Insects. 2020 Dec 29;12(1):18. doi: 10.3390/insects12010018 (PMC7823957; doi:10.3390/insects12010018)
Supplement: Supplementary file 1 [file insects-12-00018-s001.zip › insects-984553-s-XML/SUPPLEMENTARY MATERIALS_UPDATED/File S3_ANOVA results on the prevalence of infested plants.docx]

**File S3 : ANOVA results on prevalence of infested plants**

|  | numDF | denDF | F-value | p-value |
| --- | --- | --- | --- | --- |
| (Intercept) | 1 | 165 | 1060.3711 | <.0001 |
| DAS | 1 | 165 | 17.8657 | <.0001 |
| Sites | 1 | 165 | 54.0582 | <.0001 |
| Treatments | 5 | 165 | 16.8396 | <.0001 |
| DAS:Sites | 1 | 165 | 14.6847 | 0.0002 |
| DAS:Treatments | 5 | 165 | 9.7413 | <.0001 |
| Sites:Treatments | 5 | 165 | 1.5946 | 0.1643 |
| DAS:Sites:Treatments | 5 | 165 | 2.6595 | 0.0243 |

Adjohoun

$groups

Infested plants groups

Control 81.66667 a

Dezone 2 79.16667 a

Dezone 1 68.75000 ab

PlantNeem 67.50000 ab

Emacot 19 EC 55.83333 ab

Palmida soap 51.66667 b

> Emean

| DAS | Control | Palmida soap | PlantNeem | Emacot 19 EC | Dezone 2 | Dezone 1 |
| --- | --- | --- | --- | --- | --- | --- |
| 21 | 80.00000 | 80.00000 | 70 | 78.33333 | 83.33333 | 53.33333 |
| 28 | 51.66667 | 58.33333 | 45 | 31.66667 | 58.33333 | 60.00000 |
| 35 | 98.33333 | 50.00000 | 85 | 88.33333 | 88.33333 | 85.00000 |
| 42 | 96.66667 | 18.33333 | 70 | 25.00000 | 86.66667 | 76.66667 |

> Esd

| DAS | Control | Palmida soap | PlantNeem | Emacot 19 EC | Dezone 2 | Dezone 1 |
| --- | --- | --- | --- | --- | --- | --- |
| 21 | 9.813068 | 11.863420 | 10.000000 | 11.013460 | 5.773503 | 10.88662 |
| 28 | 15.244914 | 8.766519 | 7.391186 | 14.497765 | 12.583057 | 15.15354 |
| 35 | 1.666667 | 12.322818 | 10.671874 | 7.876359 | 4.194352 | 10.67187 |
| 42 | 3.333333 | 7.876359 | 16.666667 | 5.000000 | 4.714045 | 11.38550 |

N’Dali

$groups

Infested plants groups

Control 70.41667 a

Dezone 2 49.16667 b

Dezone 1 38.12500 b

PlantNeem 37.70833 b

Palmida soap 36.66667 b

Emacot 19 EC 28.75000 b

> Emean

| DAS | Control | Palmida soap | PlantNeem | Emacot 19 EC | Dezone 2 | Dezone 1 |
| --- | --- | --- | --- | --- | --- | --- |
| 21 | 50.83333 | 56.666667 | 63.33333 | 63.333333 | 73.33333 | 57.50000 |
| 28 | 82.50000 | 51.666667 | 45.83333 | 28.333333 | 61.66667 | 52.50000 |
| 35 | 77.50000 | 30.000000 | 30.00000 | 20.000000 | 32.50000 | 25.83333 |
| 42 | 70.83333 | 8.333333 | 11.66667 | 3.333333 | 29.16667 | 16.66667 |

> Esd

| DAS | Control | Palmida soap | PlantNeem | Emacot 19 EC | Dezone 2 | Dezone 1 |
| --- | --- | --- | --- | --- | --- | --- |
| 21 | 10.217541 | 2.357023 | 9.3293642 | 5.270463 | 4.906534 | 6.718548 |
| 28 | 3.154949 | 5.181877 | 5.5067299 | 3.967460 | 3.191424 | 3.695593 |
| 35 | 2.846375 | 7.817360 | 4.9065338 | 5.931710 | 3.695593 | 3.695593 |
| 42 | 4.383259 | 1.666667 | 0.9622504 | 1.360828 | 4.166667 | 5.931710 |
