# Supplementary material for: Bio-Efficacy of Diatomaceous Earth, Household Soaps, and Neem Oil against Spodoptera frugiperda (Lepidoptera: Noctuidae) Larvae in Benin
Source: Insects. 2020 Dec 29;12(1):18. doi: 10.3390/insects12010018 (PMC7823957; doi:10.3390/insects12010018)
Supplement: Supplementary file 1 [file insects-12-00018-s001.zip › insects-984553-s-XML/SUPPLEMENTARY MATERIALS_UPDATED/File S4_ANOVA results on the percentage of damaged plants.docx]

**File S4 : ANOVA results on percentage of damaged plants**

numDF denDF F-value p-value

(Intercept) 1 165 1797.7158 <.0001

DAS 1 165 12.6143 0.0005

Sites 1 165 59.5173 <.0001

Treatments 5 165 17.6530 <.0001

DAS:Sites 1 165 13.0711 0.0004

DAS:Treatments 5 165 6.3263 <.0001

Sites:Treatments 5 165 3.3314 0.0068

DAS:Sites:Treatments 5 165 3.1773 0.0091

Adjohoun

> S1=SNK.test(mod1,'Treatments');S1

$statistics

MSerror Df Mean CV

419.4444 90 81.25 25.20658

$parameters

test name.t ntr alpha

SNK Treatments 6 0.05

$snk

Table CriticalRange

2 2.809582 14.38530

3 3.370214 17.25579

4 3.701969 18.95440

5 3.936963 20.15759

6 4.118234 21.08571

$means

Damaged plants std r Min Max Q25 Q50

Control 90.83333 16.84351 16 40.00000 100 85.00000 100.00000

Dezone 1 80.00000 21.77324 16 40.00000 100 60.00000 90.00000

Dezone 2 86.25000 17.12157 16 46.66667 100 80.00000 93.33333

Emacot 19 EC 79.16667 2 4.69068 16 26.66667 100 71.66667 86.66667

Palmida soap 68.33333 21.29163 16 26.66667 100 56.66667 73.33333

PlantNeem 82.91667 20.06932 16 46.66667 100 66.66667 90.00000

Q75

Control 100.00000

Dezone 1 100.00000

Dezone 2 100.00000

Emacot 19 EC 100.00000

Palmida soap 81.66667

PlantNeem 100.00000

$groups

Damaged plants (%) groups

Control 90.83333 a

Dezone 2 86.25000 ab

PlantNeem 82.91667 ab

Dezone 1 80.00000 ab

Emacot 19 EC 79.16667 ab

Palmida soap 68.33333 b

N’Dali

> S1=SNK.test(mod1,'Treatments');S1

$statistics

MSerror Df Mean CV

356.1574 90 57.56944 32.78151

$parameters

test name.t ntr alpha

SNK Treatments 6 0.05

$snk

Table CriticalRange

2 2.809582 13.25570

3 3.370214 15.90078

4 3.701969 17.46601

5 3.936963 18.57472

6 4.118234 19.42996

$means

Damaged plants std r Min Max Q25 Q50

Control 82.91667 14.08309 16 36.666667 96.66667 80.00000 85.00000

Dezone 1 59.37500 20.55593 16 26.666667 93.33333 43.33333 63.33333

Dezone 2 65.20833 13.71502 16 46.666667 93.33333 55.83333 61.66667

Emacot 19 EC 39.16667 25.97720 16 6.666667 96.66667 20.00000 35.00000

Palmida soap 45.41667 19.88392 16 3.333333 73.33333 29.16667 48.33333

PlantNeem 53.33333 16.05546 16 30.000000 90.00000 45.83333 50.00000

Q75

Control 88.33333

Dezone 1 74.16667

Dezone 2 73.33333

Emacot 19 EC 53.33333

Palmida soap 60.83333

PlantNeem 60.00000

$groups

Damaged plants (%) groups

Control 82.91667 a

Dezone 2 65.20833 b

Dezone 1 59.37500 bc

PlantNeem 53.33333 bcd

Palmida soap 45.41667 cd

Emacot 19 EC 39.16667 d
