# Supplementary material for: Bio-Efficacy of Diatomaceous Earth, Household Soaps, and Neem Oil against Spodoptera frugiperda (Lepidoptera: Noctuidae) Larvae in Benin
Source: Insects. 2020 Dec 29;12(1):18. doi: 10.3390/insects12010018 (PMC7823957; doi:10.3390/insects12010018)
Supplement: Supplementary file 1 [file insects-12-00018-s001.zip › insects-984553-s-XML/SUPPLEMENTARY MATERIALS_UPDATED/File S6_Kruskal-Wallis test performed on phytotoxicity scores.docx]

**File S6 : Kruskal-Wallis test performed on phytotoxicity scores**

**Treatments**

Kruskal-Wallis chi-square = 577.12, df = 5, p-value < 2.2e-16

**Sites**

Kruskal-Wallis chi-square = 8.4945, df = 1, p-value = 0.003562

**Interaction Sites and Treatments**

Kruskal-Wallis chi-square= 602.6, df = 11, p-value < 2.2e-16

**### Dunn tests**

N'Dali

# A tibble: 6 x 6

Treatment count mean sd median IQR

<fct> <int> <dbl> <dbl> <dbl> <dbl>

1 Dezone 1 480 0 0 0 0

2 Dezone 2 480 0 0 0 0

3 Emacot 19 EC 480 0 0 0 0

4 PlantNeem 480 0 0 0 0

5 Palmida soap 480 0.260 0.518 0 0

6 Control 480 0 0 0 0

Dezone 1 Dezone 2 Emacot 19 EC PlantNeem Palmida soap Control

"a" "a" "a" "a" "b" "a"

Adjohoun

# A tibble: 6 x 6

Treatment count mean sd median IQR

<fct> <int> <dbl> <dbl> <dbl> <dbl>

1 Dezone 1 240 0 1.50 2 3

2 Dezone 2 240 0 1.45 3 2

3 Emacot 19 EC 240 0 1.43 2 2

4 PlantNeem 240 0 1.36 3 2

5 Palmida soap 240 0.167 1.35 2 3

6 Control 240 0 1.36 4 2

Dezone 1 Dezone 2 Emacot 19 EC PlantNeem Palmida soap Control

"a" "a" "a" "a" "b" "a"
