# Supplementary material for: Bio-Efficacy of Diatomaceous Earth, Household Soaps, and Neem Oil against Spodoptera frugiperda (Lepidoptera: Noctuidae) Larvae in Benin
Source: Insects. 2020 Dec 29;12(1):18. doi: 10.3390/insects12010018 (PMC7823957; doi:10.3390/insects12010018)
Supplement: Supplementary file 1 [file insects-12-00018-s001.zip › insects-984553-s-XML/SUPPLEMENTARY MATERIALS_UPDATED/File S7_ANOVA results on growth parameters.docx]

**File S7 : ANOVA results on growth parameters**

**Plant height**

numDF denDF F-value p-value

(Intercept) 1 1065 2310.4694 <.0001

Sites 1 1065 109.4818 <.0001

Treatments 5 1065 15.3564 <.0001

Sites:Treatments 5 1065 8.8651 <.0001

**Stem thickness**

numDF denDF F-value p-value

(Intercept) 1 1065 1192.5164 <.0001

Sites 1 1065 37.0619 <.0001

Treatments 5 1065 18.0975 <.0001

Sites:Treatments 5 1065 11.6666 <.0001

**Leaf number**

Df Deviance Resid. Df Resid. Dev Pr(>Chi)

NULL 1079 271.30

Sites 1 2.6977 1078 268.60 0.1005

Treatments 5 7.4507 1073 261.15 0.1892

Sites:Treatments 5 6.4561 1068 254.69 0.2643

**Multiple comparison tests**

Adjohoun

**Plant height**

S1=SNK.test(mod1,'Treatments');S1

$statistics

MSerror Df Mean CV

0.09385955 354 1.595306 19.20416

$parameters

test name.t ntr alpha

SNK Treatments 6 0.05

$snk

Table CriticalRange

2 2.781317 0.1100054

3 3.328513 0.1316479

4 3.650475 0.1443820

5 3.877618 0.1533659

6 4.052287 0.1602743

$means

Plant height std r Min Max Q25 Q50 Q75

Dezone 1 1.630833 0.2853917 60 0.95 2.10 1.5000 1.65 1.8500

Dezone 2 1.584167 0.3367959 60 0.75 2.25 1.4500 1.60 1.7625

Emacot 19 EC 1.708333 0.2526414 60 1.15 2.25 1.5000 1.70 1.9000

PlantNeem 1.510000 0.3693558 60 0.50 2.25 1.2875 1.55 1.7500

Palmida soap 1.520167 0.2587027 60 1.00 2.20 1.3500 1.50 1.6525

Control 1.618333 0.3179605 60 0.80 2.15 1.4375 1.65 1.8625

$groups

Plant height groups

Emacot 19 EC 1.708333 a

Dezone 1 1.630833 ab

Control 1.618333 ab

Dezone 2 1.584167 ab

Palmida soap 1.520167 b

PlantNeem 1.510000 b

**Stem thickness**

> mod2=lm(Stem thickness ~Treatments)

> S2=SNK.test(mod2,'Treatments');S2

$statistics

MSerror Df Mean CV

0.9281327 354 2.954028 32.61298

$parameters

test name.t ntr alpha

SNK Treatments 6 0.05

$snk

Table CriticalRange

2 2.781317 0.3459233

3 3.328513 0.4139803

4 3.650475 0.4540239

5 3.877618 0.4822747

6 4.052287 0.5039989

$means

Stem thickness std r Min Max Q25 Q50 Q75

Dezone 1 3.451667 1.3419239 60 2.0 6.60 2.5 2.900 3.6250

Dezone 2 3.250000 1.3293301 60 1.2 6.20 2.4 2.875 3.8500

Emacot 19 EC 2.872500 0.5246811 60 1.4 4.25 2.5 2.900 3.2000

PlantNeem 3.170000 1.0994297 60 1.0 6.00 2.5 3.000 3.5000

Palmida soap 2.550000 0.4772804 60 1.4 4.25 2.3 2.500 2.8250

Control 2.430000 0.5376660 60 1.2 3.90 2.0 2.500 2.7625

$groups

Stem thickness groups

Dezone 1 3.451667 a

Dezone 2 3.250000 ab

PlantNeem 3.170000 ab

Emacot 19 EC 2.872500 bc

Palmida soap 2.550000 cd

Control 2.430000 d

N'Dali

**Plant height**

> mod1=lm(Plant height ~Treatments)

> S1=SNK.test(mod1,'Treatments');S1

$statistics

MSerror Df Mean CV

0.05964584 714 1.770556 13.79369

$parameters

test name.t ntr alpha

SNK Treatments 6 0.05

$snk

Table CriticalRange

2 2.776514 0.06190124

3 3.321432 0.07404995

4 3.641729 0.08119085

5 3.867536 0.08622513

6 4.041078 0.09009417

$means

Plant height std r Min Max Q25 Q50 Q75

Dezone 1 1.892750 0.2522602 120 0.95 2.55 1.7225 1.900 2.0500

Dezone 2 1.887083 0.2661187 120 1.15 2.80 1.7000 1.900 2.0625

Emacot 19 EC 1.753833 0.2480552 120 1.20 2.35 1.5875 1.750 1.9500

PlantNeem 1.754833 0.2517701 120 0.50 2.40 1.6000 1.785 1.9000

Palmida soap 1.711167 0.2161628 120 1.05 2.20 1.5500 1.700 1.8500

Control 1.623667 0.2275405 120 0.90 2.25 1.5000 1.625 1.7500

$groups

Plant height groups

Dezone 1 1.892750 a

Dezone 2 1.887083 a

PlantNeem 1.754833 b

Emacot 19 EC 1.753833 b

Palmida soap 1.711167 b

Control 1.623667 c

**Stem thickness**

> mod2=lm(Stem thickness ~Treatments)

> S2=SNK.test(mod2,'Treatments');S2

$statistics

MSerror Df Mean CV

0.1797964 714 2.702639 15.68926

$parameters

test name.t ntr alpha

SNK Treatments 6 0.05

$snk

Table CriticalRange

2 2.776514 0.1074731

3 3.321432 0.1285657

4 3.641729 0.1409638

5 3.867536 0.1497043

6 4.041078 0.1564217

$means

Stem thickness std r Min Max Q25 Q50 Q75

Dezone 1 1 2.729167 0.3860260 120 1.65 3.60 2.5500 2.725 3.0000

Dezone 2 2.810833 0.3744005 120 1.55 3.80 2.6000 2.800 3.0500

Emacot 19 EC 2.651667 0.4784838 120 1.35 3.65 2.4000 2.750 2.9625

PlantNeem 2.780417 0.3981204 120 1.65 3.65 2.5500 2.775 3.0500

Palmida soap 2.795417 0.4832486 120 1.60 4.20 2.4500 2.850 3.0625

Control 2.448333 0.4106223 120 1.35 3.75 2.1375 2.450 2.7000

$groups

Stem thickness groups

Dezone 2 2.810833 a

Palmida soap 2.795417 a

PlantNeem 2.780417 a

Dezone 1 2.729167 ab

Emacot 19 EC 2.651667 b

Control 2.448333 c
