# Supplementary material for: Bio-Efficacy of Diatomaceous Earth, Household Soaps, and Neem Oil against Spodoptera frugiperda (Lepidoptera: Noctuidae) Larvae in Benin
Source: Insects. 2020 Dec 29;12(1):18. doi: 10.3390/insects12010018 (PMC7823957; doi:10.3390/insects12010018)
Supplement: Supplementary file 1 [file insects-12-00018-s001.zip › insects-984553-s-XML/SUPPLEMENTARY MATERIALS_UPDATED/File S8_ANOVA results on maize cob and grain yields.docx]

**File S8 :** **ANOVA results on maize cob and grain yields**

**Maize cob yield**

numDF denDF F-value p-value

(Intercept) 1 81 114.51684 <.0001

Sites 1 81 58.51500 <.0001

Treatments 5 81 10.93346 <.0001

Sites:Treatments 5 81 0.86394 0.5091

**Maize grain yield**

numDF denDF F-value p-value

(Intercept) 1 81 95.38088 <.0001

Sites 1 81 78.21879 <.0001

Treatments 5 81 10.86580 <.0001

Sites:Treatments 5 81 0.73282 0.6009

Multiple comparison tests

**Maize Yields**

Adjohoun

Maize cob

> sc=SNK.test(mod,'Treatments');sc

$statistics

MSerror Df Mean CV

2038682 18 5287.083 27.0059

$parameters

test name.t ntr alpha

SNK Treatments 6 0.05

$snk

Table CriticalRange

2 2.971152 2121.142

3 3.609304 2576.726

4 3.996978 2853.491

5 4.276293 3052.897

6 4.494420 3208.621

$means

Maize cob std r Min Max Q25 Q50 Q75

Control 3537.5 643.9656 4 2900 4260 3050.0 3495 3982.5

Dezone 1 5927.5 1144.6797 4 5010 7580 5265.0 5560 6222.5

Dezone 2 5297.5 1905.7348 4 3100 7650 4307.5 5220 6210.0

Emacot 19 EC 7142.5 1066.8450 4 5600 7910 6845.0 7530 7827.5

PlantNeem 4842.5 2063.4982 4 2440 6980 3520.0 4 975 6297.5

Palmida soap 4975.0 1216.1826 4 4020 6670 4132.5 4605 5447.5

$groups

Maize cob groups

Emacot 19 EC 7142.5 a

Dezone 1 5927.5 ab

Dezone 2 5297.5 ab

Palmida soap 4975.0 ab

PlantNeem 4842.5 ab

Control 3537.5 b

**Maize grain**

> sg=SNK.test(mog,'Treatments');sg

$statistics

MSerror Df Mean CV

1548039 18 3865 32.19152

$parameters

test name.t ntr alpha

SNK Treatments 6 0.05

$snk

Table CriticalRange

2 2.971152 1848.357

3 3.609304 2245.352

4 3.996978 2486.524

5 4.276293 2660.286

6 4.494420 2795.983

$means

Maize grain std r Min Max Q25 Q50 Q75

Control 2060.0 421.7424 4 1620 2560 1770.0 2030 2320.0

Dezone 1 4407.5 714.9534 4 3670 5380 4067.5 4290 4630.0

Dezone 2 3967.5 1847.6720 4 1550 6000 3275.0 4160 4852.5

Emacot 19 EC 5307.5 1129.6718 4 3910 6240 4630.0 5540 6217.5

PlantNeem 3617.5 1652.6822 4 1620 5120 2587.5 3865 4895.0

Palmida soap 3830.0 1085.2649 4 2670 5210 3187.5 3720 4362.5

$groups

Maize grain groups

Emacot 19 EC 5307.5 a

Dezone 1 4407.5 ab

Dezone 2 3967.5 ab

Palmida soap 3830.0 ab

PlantNeem 3617.5 ab

Control 2060.0 b

N’Dali

**Maize cob**

> sc=SNK.test(mod,'Treatments');sc

$statistics

MSerror Df Mean CV

3607395 66 7720.694 24.6003

$parameters

test name.t ntr alpha

SNK Treatments 6 0.05

$snk

Table CriticalRange

2 2.823568 1548.120

3 3.390864 1859.159

4 3.727463 2043.711

5 3.966331 2174.679

6 4.150851 2275.848

$means

Maize cob std r Min Max Q25 Q50 Q75

Control 5717.500 1761.823 12 3210 8310 4557.5 5865 6687.5

Dezone 1 9002.500 2118.074 12 5970 13350 7752.5 9090 9927.5

Dezone 2 8073.333 1383.857 12 6500 11560 7090.0 8075 8465.0

Emacot 19 EC 8311.667 2394.326 12 3320 11670 7342.5 8225 9955.0

PlantNeem 7163.333 1794.640 12 3430 9810 6907.5 7255 8012.5

Palmida soap 8055.833 1784.802 12 4360 10350 7670.0 8210 9307.5

$groups

Maize cob groups

Dezone 1 9002.500 a

Emacot 19 EC 8311.667 a

Dezone 2 8073.333 a

Palmida soap 8055.833 a

PlantNeem 7163.333 ab

Control 5717.500 b

**Maize grain**

sg=SNK.test(mog,'Treatments');sg

$statistics

MSerror Df Mean CV

2729798 66 6297.5 26.23597

$parameters

test name.t ntr alpha

SNK Treatments 6 0.05

$snk

Table CriticalRange

2 2.823568 1346.706

3 3.390864 1617.279

4 3.727463 1777.821

5 3.966331 1891.749

6 4.150851 1979.756

$means

Maize grain std r Min Max Q25 Q50 Q75

Control 4611.667 1506.675 12 2430 6820 3580.0 4865 5390.0

Dezone 1 7386.667 1820.876 12 4750 11040 6452.5 7450 8157.5

Dezone 2 6711.667 1204.241 12 5330 9610 5825.0 6685 7250.0

Emacot 19 EC 6694.167 2110.474 12 2750 9440 5795.0 6990 8067.5

PlantNeem 5804.167 1598.985 12 2460 8250 5585.0 5925 6497.5

Palmida soap 6576.667 1527.115 12 3550 8540 6057.5 6825 7562.5

$groups

Maize grain groups

Dezone 1 7386.667 a

Dezone 2 6711.667 a

Emacot 19 EC 6694.167 a

Palmida soap 6576.667 a

PlantNeem 5804.167 ab

Control 4611.667 b
